# Refractory Case of Takayasu Arteritis in a Young Woman: A Clinical Challenge

**DOI:** 10.7759/cureus.872

**Published:** 2016-11-09

**Authors:** Mudassar Ahmed, Salman Mansoor, Salman Assad, Shahar Y Khan, Rizwanullah Khan, Usman Ghani, Taimur Mansoor, Aasim Rehman

**Affiliations:** 1 Department of Internal Medicine, Shifa International Hospital, Islamabad, Pakistan; 2 Department of Neurology, Shifa International Hospital, Islamabad, Pakistan; 3 Department of Medicine, Shifa Tameer-e-Millat University, Islamabad, Pakistan; 4 Department of Nephrology, Shifa International Hospital, Islamabad, Pakistan; 5 Department of Medicine, Shifa International Hospital, Islamabad, Pakistan; 6 School of Medicine, Shifa College of Medicine

**Keywords:** autoimmunity, immunosupressive, vasculitis

## Abstract

Takayasu arteritis (TA) is an idiopathic chronic inflammatory vasculitis of the aorta and its main branches, which if not treated can lead to severe vascular damage and fatal vascular events. Glucocorticoids (GCs) are the mainstay of the therapy of TA but a significant proportion of patients tend to experience flare-ups when their GCs are tapered. We report a case of a 42-year-old female with TA, diagnosed according to the 1990 American College of Rheumatology Criteria for TA. Cardiovascular assessment showed normal carotid upstrokes with bilateral carotid bruits and soft right and left subclavian bruits with weak peripheral pulses. A computed tomography (CT) aortogram of the chest showed severe stenosis of bilateral subclavian arteries and mild stenosis of right and left common carotid arteries at the origin. A CT aortogram of the abdomen showed an occluded left renal artery, a very small left kidney, and mild narrowing of the abdominal aorta below the level of renal arteries.

She was initially managed with GCs along with immunosuppressive therapy including methotrexate, azathioprine, and cyclophosphamide, but her disease remained active. She was then sequentially treated with inhibitor etanercept (ETN), inhibitor tocilizumab (TCZ) and monoclonal anti-CD20 antibody rituximab (RTX), and in spite of aggressive biologic therapy she continued to have active disease. To the best of our knowledge, this is the first case of refractory TA treated sequentially with three different biologic drugs.

## Introduction

Takayasu arteritis (TA) is an idiopathic chronic inflammatory vasculitis of the aorta and its main branches, which if not treated can lead to severe vascular damage and fatal vascular events. Glucocorticoids (GCs) are the mainstay of the therapy of TA but a significant proportion of patients tend to experience flare-ups when their GCs are tapered. Immunosuppressants like methotrexate (MTX), azathioprine (AZA), cyclosporine A (Cyc A), mycophenolate mofetil (MMF) and cyclophosphamide (CYC) have all been used in patients with TA but their results have been unsatisfactory. There have been case reports and case series with biologics including tumor necrosis factor (TNF) inhibitor etanercept (ETN), interleukin 6 (IL-6) inhibitor tocilizumab TCZ) and monoclonal anti-CD20 antibody rituximab (RTX), all of which have shown promising results, but to date there have been no standardized trials to assess their efficacy [1].

## Case presentation

**Figure 1:**
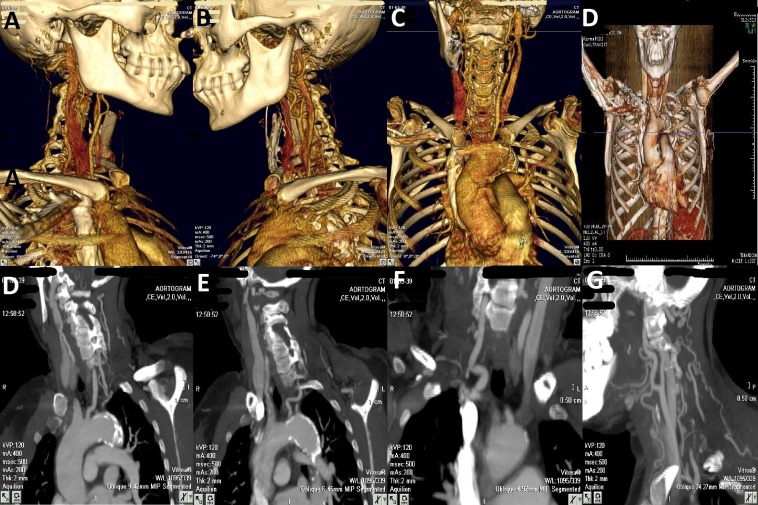
Computed tomography (CT) aortogram of chest [A-G] Severe stenosis of bilateral subclavian arteries with an appearance suggesting near occlusion. Mild to moderate stenosis of bilateral carotid arteries at the origin.

We report a case of a 42-year-old female who presented with complaints of palpitations accompanied by nausea and vomiting four years back. Her past medical history revealed that she had high blood pressure since four years. At that time she was investigated with radiological studies, serum markers and eventually diagnosed as a case of TA in accordance with the 1990 American College of Rheumatology criteria for TA [2]. An ophthalmological examination was non-contributory. The cardiovascular assessment showed normal carotid upstrokes with bilateral carotid bruits and soft right and left subclavian bruits with weak peripheral pulses. A computed tomography (CT) aortogram of the chest showed severe stenosis of bilateral subclavian arteries and mild stenosis of the right and left common carotid arteries at the origin (Figure 1).

**Figure 2:**
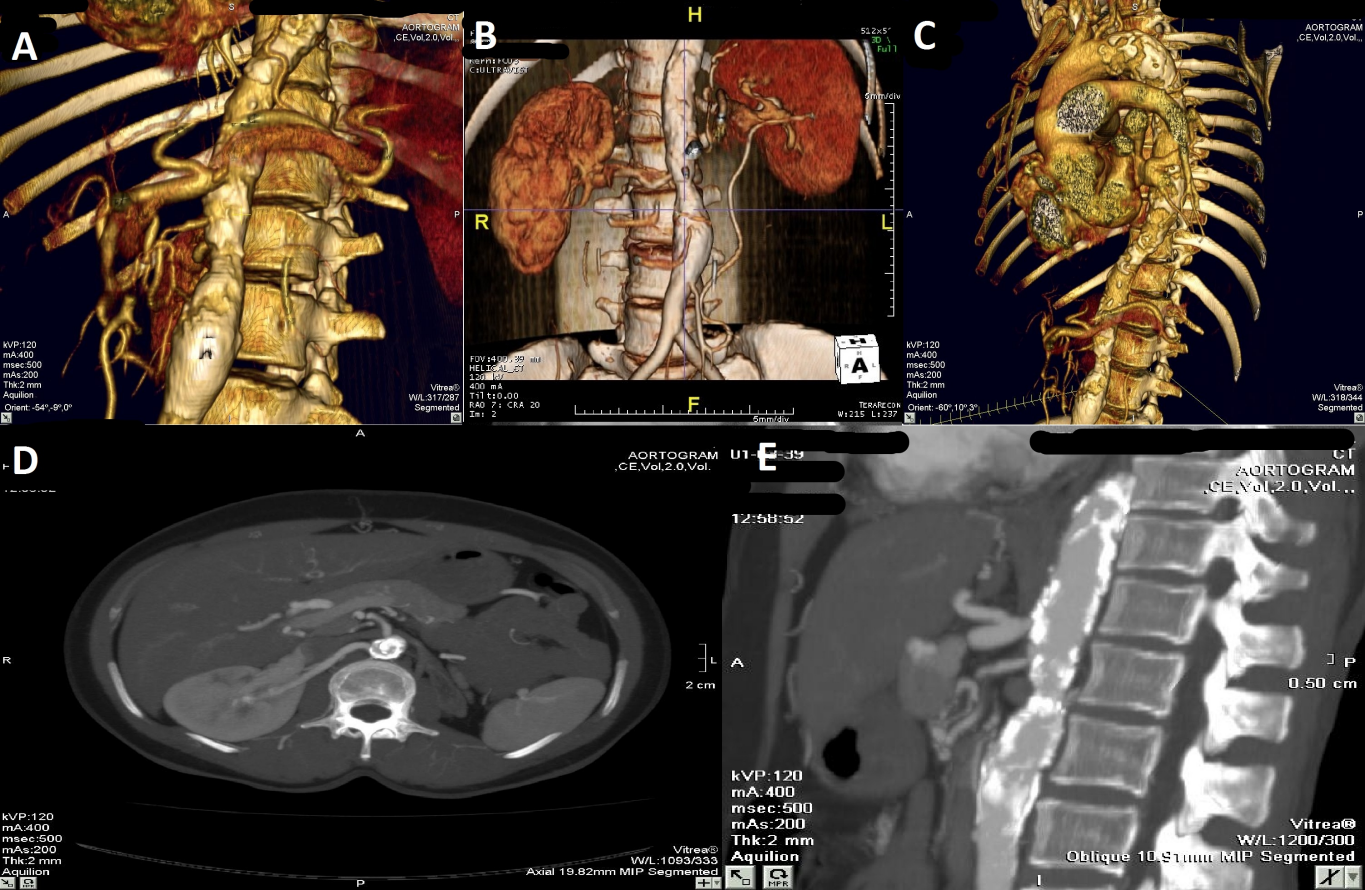
Computed tomography (CT) aortogram of abdomen [A-E] Occluded left renal artery while right renal artery is normal. The abdominal aorta is mildly narrowed at and below the level of renal arteries.

A CT aortogram of the abdomen showed an occluded left renal artery, a very small left kidney, and mild narrowing of the abdominal aorta below the level of renal arteries (Figure 2).

**Figure 3:**
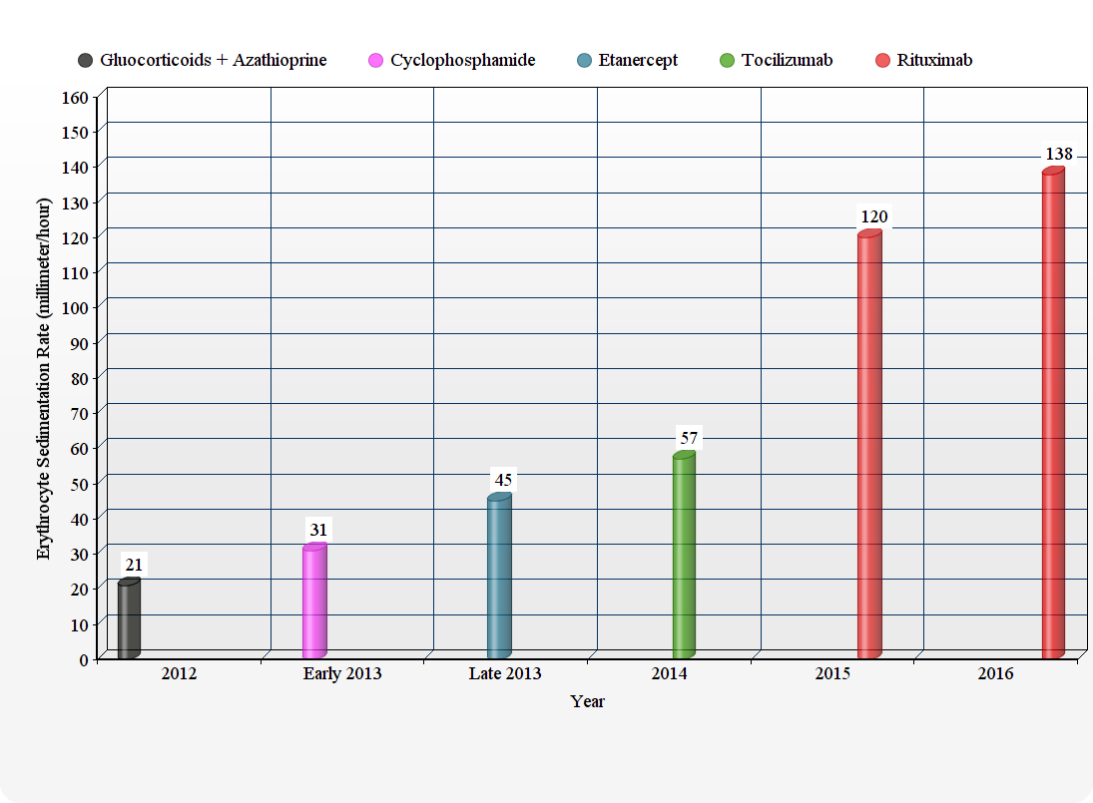
Trends of erythrocyte sedimentation rate (ESR) over the last couple of years when different treatment regimens were administered

**Figure 4:**
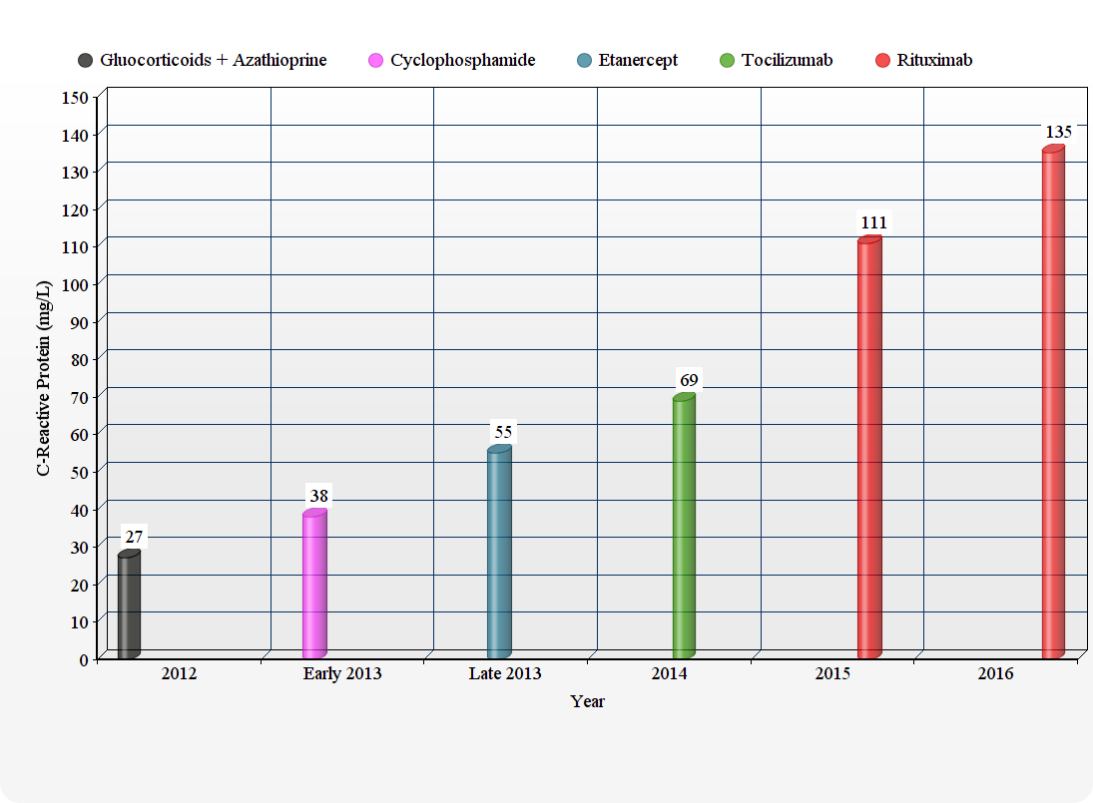
Trends of C- reactive protein (CRP) over the last couple of years when different treatment regimens were administered

She was started on a combination regimen of glucocorticoids with azathioprine. Initially, her symptoms improved for six months, but later there was a clinical decline in her condition. She was switched to cyclophosphamide. During this period, her serum erythrocyte sedimentation rate (ESR) and C- reactive protein (CRP) were regularly followed, but as it can be seen in the graphical presentation, except the initial down bulging in 2012 on glucocorticoid and azathioprine regime, it didn’t show remissive response to any regime after 2012 (Figures 3-4).

After six months of follow-up, she was put on etanercept (TNF inhibitor). During her follow-up, her routine radiological imaging and other workup for systemic review was done, but in spite of aggressive biologic therapy, she continued to have active disease. Later in her disease process, she was also put on tocilizumab (humanized monoclonal antibody against the interleukin-6 receptor) and rituximab (chimeric monoclonal antibody against the protein CD20). All these regimens were nearly tried for six months or more but during her regular follow-up she didn’t respond to any of the regimens. However, the patient does not have any serious life-threatening associations with the disease process except chronic kidney disease (serum creatinine levels 3.38 mg/dL). During her treatment process, she was once hospitalized for acute gastroenteritis. Informed consent was obtained from the patient for this study.

## Discussion

Treatment response of Takayasu arteritis (TA) is variable and a large number of patients lie in the category where they do not respond to GC and anti-TNF or even newer regimes of ETN, TCZ, and RTX. No single regime can be preferred in the treatment of TA. However, monitoring ESR and CRP levels alone may not be a reliable method to evaluate disease progression in patients with TA and should be taken in the background of both the patient's clinical picture and the imaging [3]. Inflammatory markers are not reliable indicators of TA activity in patients on TCZ and further studies are needed to confirm these preliminary observations. Treatment of refractory Takayasu arteritis (TA) remains an unresolved clinical issue. Patients usually respond to glucocorticoid (GC) therapy, but often relapse on tapering of the GC dose. 

The use of anti-tumor necrosis factor-α (TNF-α) in patients with difficult-to-treat TA could be beneficial. Comarmond C, et al. [1] showed that 27/84 (32%) patients needed to increase the dose of anti TNF-α because of uncontrolled disease and 15/84 (18%) required a change of regimen to anti-TNF (Alpha). Glucocorticoids were tapered in 52% of the patients with TA [from 20 mg (13.1–60) to 2.5 mg (0–10) daily, at baseline and after initiation of anti-TNF drugs, respectively, p<0.0001] and discontinued in 40% of the patients. After a median follow-up of 10 months [range 3–82], 20% of the patients experienced side effects that lead to discontinuation of anti TNF-α regimen [1].

Nakaoka Y, et al. studied the efficacy of TCZ therapy in patients refractory to conventional treatment including GC [4]. In his case series, there were four patients with TA who had shown GC resistance and had received TCZ infusions (8 mg/kg) every four weeks a total of at least 24 times (range 24–51). All patients achieved good clinical response and rapid normalization of the acute-phase proteins such as C-reactive protein and serum amyloid A during the therapy with TCZ. Tombetti, et al. showed that during a median follow-up visit at 14 months, four patients of TA taking TCZ, including two nonresponders to tumor necrosis factor (TNF) inhibitors, achieved the clinical response, suggesting a nonredundant role for IL-6 in TA. Inflammatory markers normalized in all patients treated with TCZ [5]. This is in contrast to our result, where the patient did not respond to TCZ therapy and inflammatory markers along with the clinical symptoms worsened with time.

Hoyer, et al. reported accompanying immunological data implicating B-cell involvement in active TA [6]. They demonstrated raised plasmablast levels, leading to increased expression of CD19/CD20/CD27high/HLA-DR+ B cells in peripheral blood during acute exacerbations [6]. Ernst D, et al. demonstrated that four weeks after the first cycle of rituximab in TA, the prednisolone dose was successfully reduced. The 5 mg/month dose reductions of GC along with rituximab showed no evidence of relapse. For the past 12 months, the patient remained in clinical remission with a maintenance dose of 5 mg/d of GC [7]. Rituximab was given to our patient as a last resort, but unfortunately it failed to show any clinical response in our patient as evident in Figures 2-3. We need a better understanding of the pathophysiology of Takayasu arteritis (TA) to build better treatment medications. This is the first TA case in literature that showed pan-resistance to immunosuppressives, anti-TNF drugs, and monoclonal antibodies.

## Conclusions

Given the rare nature of the disease, numbers treated in individual centres will remain small, making meaningful prospective randomised studies difficult. We, therefore, propose the establishment of a registry of such patients, incorporating standardised disease staging, markers of activity and functional status to help guide future management in these patients.
